# Nonstructural Protein 11 of Porcine Reproductive and Respiratory Syndrome Virus Suppresses Both MAVS and RIG-I Expression as One of the Mechanisms to Antagonize Type I Interferon Production

**DOI:** 10.1371/journal.pone.0168314

**Published:** 2016-12-20

**Authors:** Yan Sun, Hanzhong Ke, Mingyuan Han, Ning Chen, Weihuan Fang, Dongwan Yoo

**Affiliations:** 1 Department of Pathobiology, University of Illinois at Urbana-Champaign, Urbana, Illinois, United States of America; 2 Institute of Preventive Veterinary Medicine, Zhejiang University, Hangzhou, China; Universitat Bern, SWITZERLAND

## Abstract

Type I interferons (IFN-α/β) play a key role in antiviral defense, and porcine reproductive and respiratory syndrome virus (PRRSV) is known to down-regulate the IFN response in virus-infected cells and pigs. In this study, we showed that the overexpression of nsp11 of PRRSV induced a strong suppression of IFN production. Nsp11 suppressed both IRF3 and NF-κB activities when stimulated with a dsRNA analogue and TNF-α, respectively. This suppression was RLR dependent, since the transcripts and proteins of MAVS and RIG-I, two critical factors in RLR-mediated pathway, were both found to be reduced in the presence of overexpressed nsp11. Since nsp11 is an endoribonuclease (EndoU), the structure function relationship was examined using a series of nsp11 EndoU mutant plasmids. The mutants that impaired the EndoU activity failed to suppress IFN and led to the normal expression of MAVS. Seven single amino acid substitutions (4 in subdomain A and 3 in subdomain B) plus one insertion (frame-shift in nsp11) were then introduced into PRRSV infectious cDNA clones to generate nsp11 mutant viruses. Unfortunately, all EndoU knock-out nsp11 mutant viruses appeared replication-defective and no progenies were produced. Three mutations in EndoU subdomain A expressed the N and nsp2/3 proteins but their infectivity diminished after 2 passages. Taken together, our data show that PRRSV nsp11 endoribonuclease activity is critical for both viral replication and IFN antagonism. More importantly, the endoribonuclease activity of nsp11 demonstrates the substrate specificity towards MAVS and RIG-I (transcripts and proteins) over p65 and IRF3 in the context of gene transfection and overexpression. This is likely a mechanism of nsp11 suppression of type I IFN production.

## Introduction

Type I interferons (IFN-α/β) play a key role for antiviral defense in host cells [1–3]. For RNA viruses, the viral genome is first recognized by specific receptors including toll-like receptor 3/7 (TLR-3/7) and cytosolic receptors. Retinoic acid-inducible gene I (RIG-I) or melanoma differentiation-associated gene 5 (MDA5) are the well-known sensors in the cytoplasm, and their activations will recruit TANK-binding protein-1/I-κB kinase ε (TBK-1/IKKε) and TGFβ-activated kinase-1 (TAK-1) to mitochondrial anti-viral signaling protein (MAVS; also named VISA, IPS-1), resulting in the phosphorylation of interferon regulatory factor 3 (IRF3) and subunits of the nuclear factor (NF)-κB [4–6]. Activated IRF3 and NF-κB are then translocated to the nucleus and form a transcriptionally competent enhanceosome along with cAMP response element-binding (CREB)-binding protein (CBP) and other transcription factors, leading to the expression of type I IFN genes [7].

Porcine reproductive and respiratory syndrome (PRRS) is a swine disease that emerged in the US and Germany independently but almost simultaneously in the late 1980s [8, 9]. PRRS has since quickly spread globally and has become one of the most economically significant diseases to the pork industry worldwide. The causative agent is the PRRS virus (PRRSV) in the family *Arteriviridae* that forms the order *Nidovirales* along with two other families, *Coronaviridae* and *Roniviridae*. The PRRSV genome is a single-strand positive-sense RNA containing 10 open reading frames (ORFs): ORF1a, ORF1b, ORF2a, ORF2b, ORF3 up to ORF7, plus the newly identified ORF5a [10, 11]. PRRS affects breeding herd and grower/finisher pigs, manifesting reproductive problems including abortions and foetal deaths in sow, increased mortality in neonates, and respiratory problems in young pigs. PRRSV infection causes the suppression of type IFN induction in alveolar macrophages and in lungs of pigs where PRRSV actively replicates [12, 13]. Several viral proteins in PRRSV have been shown to downregulate IFN production, including nsp1, nsp2, nsp4, nsp11, and N [14–19]. Among them, nsp11 is the only one harboring RNA nuclease activity. Although its IFN antagonism has been previously reported (including by our lab) and its crystal structure has been recently solved, the mechanism of how it inhibits type I IFN production remains unknown [20–25].

Nsp11 is produced from a large polyprotein, PP1ab, translated directly from the viral genome, PP1ab. It is a 223 amino acids protein containing a highly conserved EndoU domain unique for viruses in the order Nidovirales. The EndoU domain resides in the C-terminal region in PRRSV nsp11 and shows a distant relationship with the XendoU family. XendoU is an endoribonuclease derived from Xenopus laevis [26–28]. PRRSV nsp11 has the uridylate-preference cleavage of RNA and consists of two subdomains, subdomain A and subdomain B. Subdomain A is thought to maintain the nuclease activity while subdomain B may be important for overall structural conformation. From its newly determined crystal structure, nsp11 assembles into an asymmetric dimmer [22], which differs from the hexametric structure of coronaviruses nsp15 [29, 30]. However, enzymatic sites of PRRSV nsp11 are perfectly superimposed on coronavirus nsp15 with two His residues in the “active loop” and one Val residue and one Thr residue in the “supportive loop” [31–34]. In addition, nsp11 in equine arteritis virus (EAV), which structurally mimics PRRSV nsp11, is reported to be essential for viral replication [35].

In the present study, we show that nsp11-induced IFN-β suppression is mediated by the inhibition of retinoic acid-inducible gene-1-like receptor (RLR)-mediated pathways. Specifically, the protein and mRNA level of both MAVS and RIG-I, but not p65 and IRF3, were reduced when exposed to the overexpressed nsp11 in cells, arguing its limited restriction of targets. The transfection of nsp11 mutants that lost the EndoU activity was unable to reduce MAVS mRNA and exhibited normal IFN production. Unfortunately, EndoU knock-out mutant genomic clones were unable to generate infectious progeny. However, we observed the reduced expression of both RIG-I and MAVS during WT PRRSV infection, suggesting nsp11 may preserve the same function during viral infection. Taken together, our data show that the PRRSV EndoU activity plays an essential role for viral replication and participates in innate immune modulation.

## Results

### Suppression of IFN-β production by nsp11

**Fig 1:**
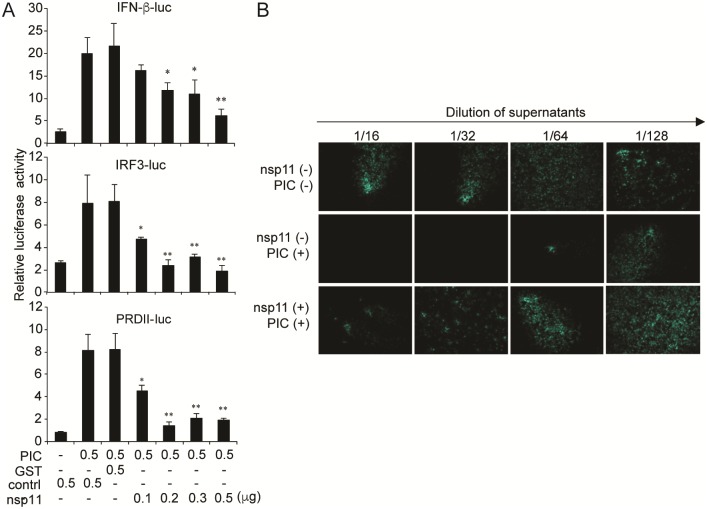
Suppression of IFN-β production by nsp11. (A) MARC-145 cells were co-transfected with 0.05 μg of pRL-TK, an indicated amount of nsp11, or 0.5 μg of GST along with 0.5 μg of pIFN-β-luc, p4xIRF3-luc, or p(PRDII)_2_-luc. At 24 h post-transfection, cells were transfected with poly(I:C) (0.5 μg/ml) for 16 h, and luciferase activities were measured using the Dual Luciferase assay system according to the instructions (Promega). The reporter plasmid containing the firefly luciferase gene placed under the target gene promoter and the Renilla luciferase vector, referred to as pRL-TK, were co-transfected into cells. The Renilla plasmid served as an internal control to normalize the experimental variability including transfection efficiencies, different numbers of cells, loading differences, and cell lysis efficiency. Cell lysates were prepared and the firefly luciferase activity was first determined (number “a”). This reaction was quenched and the renilla luciferase activity was measured (number “b”). Relative luciferase activity was then calculated by a ratio of a/b. In Fig 1A, the relative luciferase activity was only normalized with the internal Renilla control. The basal level in MARC-145 cells was considered to be the ratio in samples transfected with the vector control. The experiments were conducted in duplicate and repeated three times for statistical analysis. Each sample from nsp11-transfected cells was compared to poly(I:C) stimulation (*, P<0.05; **, P<0.01 analyzed by one-way ANOVA and bonferroni multiple comparison). (B) Suppression of IFN-β production by PRRSV nsp11 as determined by IFN bioassay using VSV-GFP. The nsp11-gene was transfected into HeLa cells for 24 h to allow its expression, followed by either mock- or poly(I:C)-transfection. Cell culture supernatants were collected 8 h after poly(I:C) transfection and were diluted in PBS to make two-fold serial dilutions. MARC-145 cells were freshly grown in 96-well plates and incubated for 24 h with each dilution of the culture supernatants, followed by infection with VSV-GFP at an MOI of 0.1. Cells were further incubated for 16 h and the GFP expression were visualized under microscope.

Many viruses express viral proteins that degrade cellular proteins or bind to cellular RNA to avoid IFN induction and antiviral effector functions [36–39]. PRRSV nsp11 contains an IFN promoter suppressive activity but its molecular action is unknown [20, 21, 23–25]. To study this function of nsp11, MARC-145 cells were transfected with nsp11, which was Flag-tagged at the N-terminus, and the pIFN-β-luc, pIRF3-luc, or pPRDII-luc reporter plasmids followed by transfection with poly(I:C) for IFN stimulation (Fig 1A). pIRF3-luc and pPRDII-luc contain 4 copies of the IRF3 binding sequence or 2 copies of the NF-κB binding sequence located in the IFN-β promoter, respectively, in front of the luciferase reporter gene. Glutathione S-transferase (GST) was derived from the parasitic helminth *Schistosoma japonicum*, and is frequently used as a tag for detection of a protein and thus was used as a negative control in this study. While cells transfected with the empty vector (control) and non-related gene GST showed high induction of IFN activity, nsp11-expressing cells exhibited strong suppression of IFN-β, IRF3, and NF-κB activities. As the amount of nsp11 was increased, the suppression became stronger, suggesting that this activity was dose-dependent and that the suppression was through the inhibition of both IRF3 and NF-κB promoter activities. To confirm the reporter assays, an IFN bioassay was conducted using vesicular stomatitis virus expressing GFP (VSV-GFP). VSV is highly sensitive to IFN, and thus secretion of IFN in the culture supernatant can be determined by monitoring the intensity of green fluorescence during VSV replication [40]. Culture supernatants collected from nsp11-transfected HeLa cells were serially diluted by 2-folds and each diluted supernatant was then incubated with MARC-145 cells in 96-well plates, followed by infection with VSV-GFP. Poly(I:C) transfection at 0.5 μg per well in a 6-well plate stimulated the secretion of IFN to culture supernatants of cells and thus a negligible level of fluorescence was seen at 1:64 dilution of culture supernatant (Fig 1B, middle panels). In contrast, GFP expression was readily observed in cells expressing nsp11 at 1:16 dilution even after poly(I:C) stimulation (Fig 1B, bottom panel), indicating a minor amount of IFN in nsp11-overexpressing cells. These results show that nsp11 has the ability to suppress the IFN-β production pathway.

### Inhibition of RLR-mediated IFN production by overexpression of nsp11

**Fig 2:**
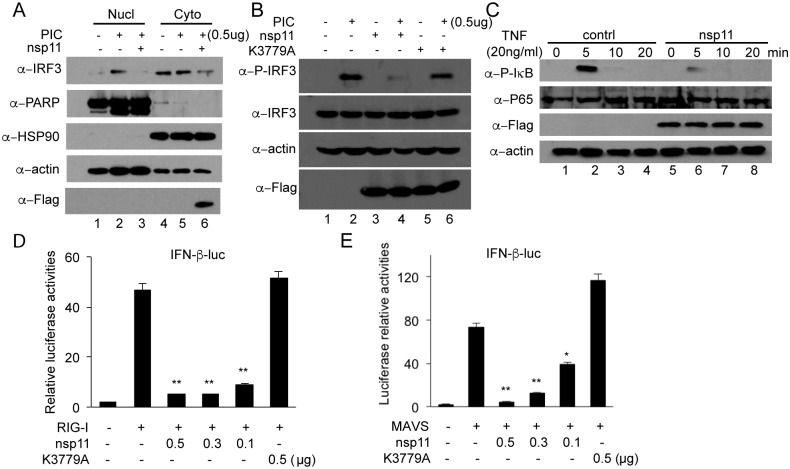
Suppression of IFN by nsp11 is via inhibition of RLR-I signaling. (A) HeLa cells were transfected with vector control or nsp11 and at 24 h post-transfection, stimulated with poly(I:C) (0.5 μg/ml) for 6 h. Cells were harvest and fractionated to nuclear and cytosolic fractions using Nuclear/Cytosol Fractionation Kit (BioVision). Both nuclear and cytosol fractions were electrophoresed by SDS-PAGE, and Western blot was conducted using α-IRF3 PAb, α-Flag MAb, α-PARP PAb, α-HSP90 MAb, and α-β-action MAb. PARP was included as a nuclear protein marker, and HSP90 was included as a cytosol protein marker. Actin was used as a loading control. Flag antibody was used to detect nsp11 expression. (B) HeLa cells were transfected with empty vector, nsp11, or K3779A, followed by stimulation with poly(I:C) (0.5 μg/ml) for 6 h. Cells were lysed and the lysates were subjected to SDS-PAGE and Western blot using rabbit α-pIRF3 PAb and rabbit α-IRF3 PAb. Rat α-FLAG MAb antibody was used to detect WT nsp11 and K3779A mutant expression. (C) HeLa cells were transfected with nsp11, and at 24 h post-transfection treated with 20 ng/ml of TNF-α for 5, 10, or 20 min. Cells were lysed at indicated time points and each lysate was subjected to SDS-PAGE and Western blot using α-nsp11 PAb, α-β-actin MAb, α-phosphor-IκB αPAb, or α-p65 PAb. Flag antibody was used to detect nsp11 expression. (D, E) MARC-145 cells were co-transfected with pIFN-β-luc (0.5 μg), pRL-TK (0.05 μg), and indicated amounts of nsp11or empty vector along with the constitutively active form of RIG-I (0.5 μg) (D) or MAVS (0.5 μg) (E). Cells were lysed at 40 h post-transfection and assayed for luciferase activities. Each set of samples were compared to the value of RIG-I or MAVS alone transfection (*, P<0.05; **, P<0.01 by one-way ANOVA and bonferroni multiple comparison).

The findings on the suppression of the IRF3 promoter activities by overexpression of nsp11 suggest that nsp11 may cause the inhibition of IRF3 activation. IRF3 normally resides in the cytoplasm in an inactive form. But upon stimulation, it undergoes phosphorylation, dimerization, and subsequent translocation to the nucleus. Thus, sub-cellular localization of IRF3 was first examined in nsp11-expressing cells. Poly(I:C) transfection caused IRF3 nuclear translocation as anticipated, whereas in nsp11-expressing cells, IRF3 was not detected in the nucleus (Fig 2A, top panel, lane 2, 3). Nsp11 expression was detected only in the cell cytoplasm using Flag antibody. This observation was confirmed by immunofluorescence of IRF3 (S1 Fig). Since IRF3 did not undergo nuclear translocation in nsp11-expressing cells, its phosphorylation state was examined. When stimulated with poly(I:C), IRF3 was effectively phosphorylated in control but was reduced in nsp11-expressing cells. This reduction was not due to the change in total amounts of IRF3 (Fig 2B, lanes 1, 2, 3, 4), indicating that the suppression of IFN induction by nsp11 was due to the inhibition of IRF3 phosphorylation.

In addition to suppressing IRF3 activity, overexpression of nsp11 was able to suppress NF-κB promoter activity, suggesting nsp11 may target the NF-κB pathway as well. One readout of NF-κB activation is p65 nuclear translocation. Therefore, we first examined the localization of p65 in the presence of transfected nsp11. The p65 subunit was normally translocated to the nucleus in control cells after stimulation (S2 Fig, bottom panel, yellow arrow). In sharp contrast, its nuclear translocation was inhibited in nsp11 overexpressing cells (S2 Fig, bottom panel, white arrows), suggesting that nsp11 blocks the p65 nuclear localization. In order for p65 to go to the nucleus, IκB needs to be phosphorylated and degraded, leading to the release of p65. We thus examined IκB phosphorylation in nsp11-expressing cells. When stimulated with TNF-α in control cells, phosphorylated IκBα was detectable at 5 min of stimulation (Fig 2C, top panel, lane 2) and degraded shortly after. By 20 min, phosphorylated IκBα was virtually undetectable, which was due to rapid degradation. In nsp11-transfected cells however, phosphorylated IκB was less abundant than in control cells, especially at 5 min of TNF-α stimulation (Fig 2C, top panel, lane 6), indicating the inhibition of IκB phosphorylation by nsp11 overexpression.

The fact that both IRF3 and NF-κB activations are inhibited by the overexpression of nsp11 implies that some common factor(s) upstream of IRF3 and NF-κB activation pathways might be hijacked by nsp11. RIG-I and MAVS are two major candidates. To examine this, we took advantage of a constitutive RIG-I signaling activator, pcDNA3-RIG-I, which contains the N-terminal CARD domain of RIG-I. When cells were co-transfected with pIFN-β-Luc and pcDNA3-RIG-I, strong IFN-β promoter activity was observed (Fig 2D). In nsp11-overexpressing cells however, the IFN activity was inhibited significantly and the inhibition was dose-dependent. Similarly, in cells overexpressing MAVS by transfection with pFlag-MAVS plasmids, the luciferase activity was increased up to 80 folds (Fig 2E), whereas in cells co-expressing nsp11, this activity was significantly reduced in a dose-dependent manner. When cells were co-transfected with the basal level promoter plasmid pTATA-Luc and nsp11, the luciferase activities remained at a basal level even when stimulated with each of the four inducers RIG-I, MAVS, poly(I:C), and IRF3 (S3 Fig), indicating the specific stimulation for IFN promoter. Taken together, nsp11 inhibits the RLR-mediated signaling and antagonizes IFN expression.

### Reduced expression of MAVS and RIG-I by overexpression of nsp11

**Fig 3:**
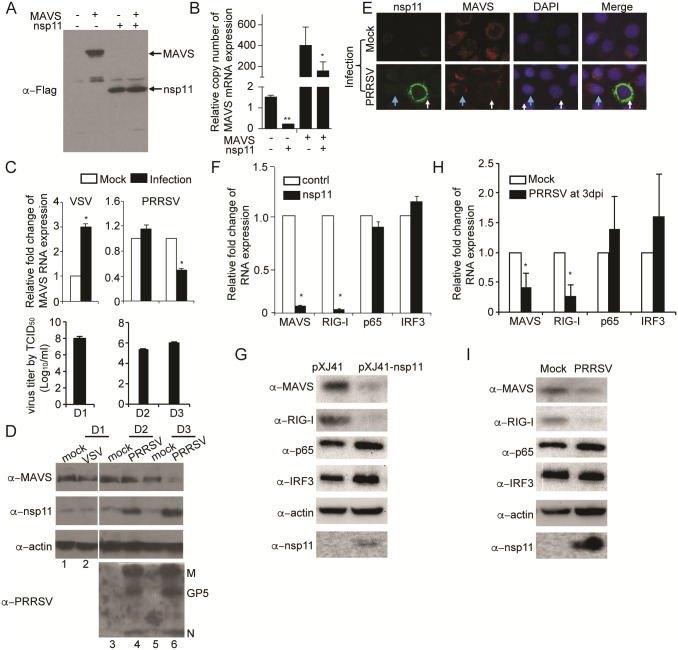
Reduced expression of MAVS and RIG-I by overexpression of nsp11. (A, B) MARC-145 cells were co-transfected with nsp11 and pFlag-MAVS. At 24 h post-transfection, total cell lysates were prepared and subjected to Western blot using anti-Flag MAb (A), or total cellular RNA was extracted for cDNA synthesis using random primers followed by qPCR in the SYBR Green system (B). For (A), lane 1, cells transfected with the empty vector only. Lane 2), cells transfected with the Flagged MAVS plasmid. Lane 3), cells transfected with wild-type Flagged nsp11 plasmid. Lane 4, cells co-transfected with the Flagged nsp11 and Flagged MAVS plasmids. (C) MARC-145 cells were infected with PRRSV or VSV-GFP at an M.O.I. of 0.1. Infected cells and supernatants were harvested at indicated times post-infection. Cells were lysed by Trizol for MAVS mRNA analysis by RT-qPCR. MAVS gene expression was shown as the fold change of copy numbers from infected cells (black bars) relative to that of uninfected cells (white bars). Titers of viruses were determined by TCID_50_ using cell culture supernatants. (D) Cell lysates were prepared in parallel and subjected to SDS-PAGE and Western blot using mouse α-MAVS PAb, rabbit α-nsp11 PAb, α-PRRSV swine serum, and α-β-actin MAb. (E) MARC-145 cells were infected with PRRSV at an M.O.I. of 5, and at 24 h post-infection, cells were stained with mouse α-MAVS PAb, rabbit α-PRRSV nsp11 PAb, and DAPI. Blue arrows indicated uninfected cells and white arrows indicated virus-infected cells. (F-I) MARC-145 cells were either transfected with nsp11 (F, G), or infected with PRRSV(H, I). At 24 h post-transfection or D3 post-infection, total cellular RNAs and cell lysates were harvested. For qPCR, cDNA were synthesized using random primers followed by qPCR using the MAVS, RIG-I, p65, and IRF3 specific primers. MAVS mRNA fold changes were calculated based on the copy numbers in nsp11-transfected cell relative to those of mock-transfected cell control. All gene copy numbers was relative to the housing keeping gene GAPDH. (n = 3, *, P<0.05; **, P<0.01, determined by 2 tailed t-test) (F, H). For protein, cell lysates were subjected to SDS-PAGE and Western blot for MAVS, RIG-I, p65, IRF3, β-actin, and PRRSV nsp11 (G, I).

Since we observed overexpressed nsp11 was able to reduce IFN promoter activities induced by both RIG-I and MAVS (Fig 2), we first examined whether MAVS function was altered by nsp11. MARC-145 cells were co-transfected with MAVS and nsp11, and total RNA and cellular lysates were individually prepared for quantitative RT-PCR and Western blot, respectively. Note that the endogenous MAVS was detected using MAVS specific antibodies (Abs), and the transfected MAVS and nsp11 were detected using Flag Abs since both MAVS and nsp11 plasmids contained Flag tag after the corresponding genes. Strikingly, exogenous MAVS protein was not detectable by Flag Abs in nsp11-expressing cells (Fig 3A), and both endogenous and exogenous MAVS mRNAs were significantly reduced in these cells (Fig 3B and S4A Fig), suggesting that nsp11 impaired the MAVS function by dampening its mRNA. Note the weak bands above nsp11 in Fig 1A were considered non-specific since they were present in all lanes.

The reduction of MAVS expression by nsp11 was validated in virus-infected cells. Vesicular stomatitis virus (VSV) was included as an nsp11-unrelated virus control. MARC-145 cells were infected with PRRSV or VSV with an MOI of 0.1, and endogenous MAVS mRNA was examined by qRT-PCR. In VSV-infected cells, the amount of MAVS mRNA was increased, showing the normal cellular response to viral infection as anticipated (Fig 3C, 3D and S4B Fig). In PRRSV-infected cells, the decrease of MAVS mRNA and protein became evident at D3 post-infection (Fig 3C and 3D). This event was rather late and we think this may be due to the slow replication and spread of PRRSV in MARC-145 cells. The viral titers for both viruses were comparable at the time of collection, indicating both viruses replicated well in MARC-145 cells (Fig 3C). To further examine the decrease of MAVS during early viral infection, MARC-145 cells were infected with PRRSV at an MOI of 5 and MAVS expression was examined by immunofluorescence at 24 h post-infection. Both nsp11 and MAVS proteins were found to localize in the perinuclear region, but MAVS was diminished in PRRSV-infected cells compared to uninfected cells (Fig 3E, See S6 Fig for more cells). Together, our data indicate that MAVS mRNA was reduced in both nsp11-gene transfected cells and PRRSV-infected cells, and the reduction of MAVS mRNA led to less protein expression, subsequently interfering with RLR signaling.

Since the level of MAVS mRNA was decreased by nsp11, it was of interest to determine whether the reduction of MAVS was specific. To do this, MARC-145 cells were transfected with nsp11 alone and mRNA/protein of MAVS, RIG-I, p65, and IRF3 were individually examined by qRT-PC/western blot. Interestingly, RIG-I mRNA was found to be significantly reduced in addition to MAVS mRNA, whereas p65 mRNA and IRF3 mRNA remained relatively unchanged (Fig 3F). Similar trend was observed for protein as well (Fig 3G). We conducted a similar experiment during PRRSV infection, and obtained a similar finding that the protein levels of MAVS and RIG-I, but not p65 and IRF3, were significantly reduced (Fig 3H and 3I). This suggests that nsp11 does not target the host transcriptome globally. So far we identified MAVS and RIG-I as the two substrates of nsp11 to antagonize type I IFN production. Note that this conclusion is drawn majorly from the transfection system and indirectly from viral infection.

### Reduction of MAVS mRNA by EndoU activity of nsp11

**Fig 4:**
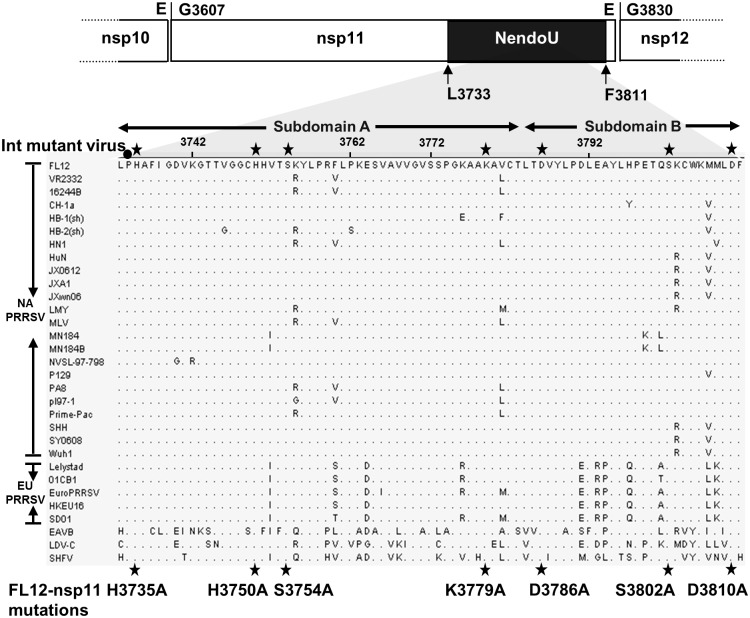
Sequence alignments of the EndoU domain of arteriviruses and positions of seven mutations. Numbers refer to amino acid positions with respect to PP1ab of PRRSV strain FL12. The PP1ab cleavage sites flanking nsp11 are indicated. PRRSV nsp11 spans between G3607 and E3829, and the EndoU domain lies from L3733 to F3811 forming the conserved 79 residue core. Subdomain A is conserved across the XendoU/EndoU family and includes 3 putative catalytic residues of H3735, H3750, and K3779. Subdomain B is specific for nidoviruses and includes two conserved Asp residues at 3786 and 3810. S3754 in subdomain A and S3802 in subdomain B are predicted to not affect the EndoU function. Stars in black indicate the amino acids targeted for mutation in the present study. A closed circle indicates the site for out-of-frame mutation (Ins-mut) in which one nucleotide of ‘C’ was inserted after the proline codon (CCC) to serve as a negative control. Thirty- one virus isolates in the family Arteriviridae were aligned representing EAV, LDV, SHFV, and PRRSV. GenBank accession numbers are shown in Material and Methods.

Since EndoU activity was previously shown to participate in the IFN antagonism of nsp11 [20, 21, 24], we wondered whether the reduction of MAVS mRNA, one of the targets of nsp11, was due to its endoribonuclease activity (EndoU). Since EndoU of EAV nsp11 is closely related to PRRSV nsp11, all our mutations was made based on the previous study on EAV nsp11 and the newly released PRRSV nsp11 crystal structure. Specifically the double mutations of H2963A and H2978A or single mutation of H3007A in EAV nsp11 subdomain A reduced the cleavage of RNA to undetectable levels [35]. Moreover, D3014 and D3038 in EAV nsp11 subdomain B were shown to be critical for both genomic and subgenomic viral RNA synthesis [35], and those mutations rendered the mutant nsp11 proteins insoluble [41]. These findings were confirmed for the SARS-CoV nsp15 protein [30, 41]. The two Asp (D) residues took part of an extensive hydrogen-bond network in the close proximity to the active site and thus replacing those residues might have caused an indirect effect on the nsp11 activity by disturbing its secondary structure. As mentioned earlier, PRRSV nsp11 enzymatic sites in subdomain A overlay with EAV and coronavirus nsp11 active sites [22]. Therefore, through alignment of EAV nsp11 and PRRSV nsp11s from North Americans strain and European strains, seven nsp11 mutants were constructed accordingly in which Ala mutations were introduced to the EndoU domain of PRRSV nsp11 as shown in Fig 4. Histidine substitutions at H3735, H3750, and K3779 in subdomain A of PRRSV nsp11, equivalent to H129, H144, and K173 indicated in nsp11 crystal structure, altered the EndoU catalytic sites. D3786 and D3810 in subdomain B, equivalent to D3014 and D3038 in EAV nsp11 and D180 and D204 in nsp11 crystal structure, were regarded to impair the secondary structure, and thus Ala mutations were introduced to these sites. Residues S2982 and S3030 did not affect any functional changes of the EndoU activity for EAV [35], and thus equivalent mutations of S3754A and S3802A were made to PRRSV nsp11 to use as negative controls.

**Fig 5:**
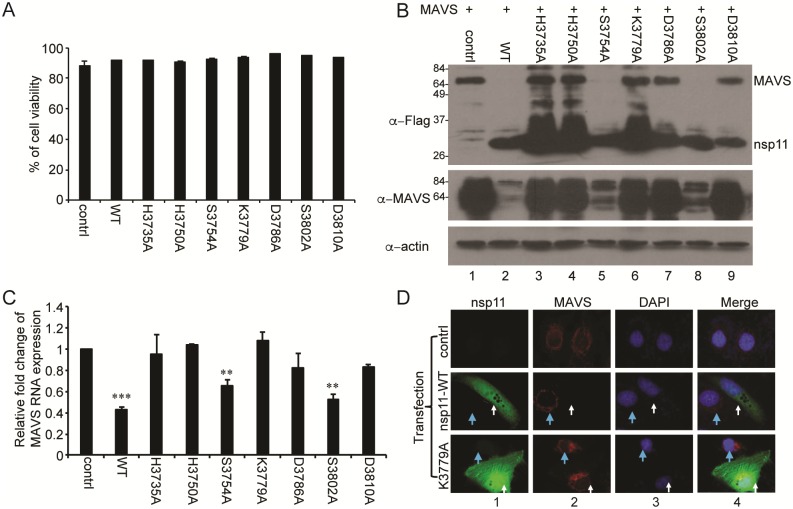
Reduction of MAVS mRNA through EndoU activity of nsp11. (A) MARC-145 cells were transfected with vector control, nsp11-WT or individual nsp11 mutants (2.0 μg/plasmid). At 24 h post-transfection, cells were trypsinized and stained with Aqua live/dead cell staining kit. Cells were applied to flow cytometry to count live cells and dead cells. Each bar represents the mean value of three individual experimental results. (B) HeLa cells were co-transfected with pFlag-MAVS and nsp11-WT or individual nsp11 EndoU mutant plasmids (0.5 μg/plasmid). At 24 h post-transfection, cells were lysed and subjected to Western blot with mouse α-Flag MAb, mouse α-MAVS PAb, and α-β-actin MAb. (C) HeLa cells were transfected with vector or individual mutant nsp11 plasmids (0.5 μg/plasmid). At 24 h post-transfection, total RNA was extracted and endogenous MAVS mRNA was quantified via qPCR. For each sample, MAVS mRNA fold change was calculated by the relative copy number of MAVS mRNA from 24 h post-transfection with respect to 0 h. Each sample expressing nsp11 or mutants was compared with empty vector-transfected sample (n = 3, *, P<0.05 by 2 tailed t-test). (D) MARC-145 cells were transfected with 0.5 μg of empty vector, nsp11, or K3779A, and at 24 h post-transfection, stained with respective antibodies as described for virus-infected cells. White arrows indicate cells expressing nsp11 or mutant nsp11 K3779A. Blue arrows indicate cells that are not expressing nsp11.

Cell cytotoxicity was of an initial concern during our study, since nsp11 is known to have cytotoxicity in some of the cell lines and it would be possible that the observation of IFN suppression could be the result of “dying cells” due to the cytotoxicity by ectopically over-expressing nsp11. Therefore, cell viability experiments were conducted using aqua Live/Dead staining followed by flow cytometry. The results indicated that nsp11 WT and mutant plasmids exhibited minimal and similar proportion of dead cells in the MARC-145 cell line (Fig 5A), supporting that the IFN suppression by nsp11 was not due to the cell cytotoxicity. We then continued to examine the reduction of MAVS expression in cells overexpressing individual nsp11 mutants. Cells were co-transfected with Flagged MAVS and individual nsp11 mutants. Since MAVS was fused with Flag, anti-Flag antibody would only detect exogenous MAVS, while MAVS PAb would detect both exogenous and endogenous MAVSs in cells. As shown in Fig 5B, nsp11-S3754A and nsp11-S3802A which were not supposed to affect the EndoU activity showed the MAVS reduction as with nsp11-WT, and all other mutants did not change the level of MAVS. Interestingly, exogenous MAVS was not detectable by anti-Flag MAb in cells expressing nsp11-WT, nsp11-S3754A, and nsp11-S3802A (Fig 5B, lanes 2, 5, 8, top panel), whereas low levels of endogenous MAVS was detectable when using MAVS PAb (Fig 5B, middle panel, lanes 2, 5, 8). To further validate the reduction of endogenous MAVS, mRNA and protein levels were examined by qRT-PCR and IFA in nsp11-mutant expressing cells. For MAVS mRNA, the mutants with impaired EndoU activity showed normal levels of MAVS expression compared to empty vector control, except D3810A (Fig 5C). D3810A was postulated to reduce MAVS likely by misfolding of the nsp11 protein. For MAVS protein, nsp11-K3779A was chosen as a representative mutant from the group of mutants that specifically lost the EndoU activity. By IFA, the MAVS signal was weak (blue arrows) in nsp11-WT expressing cells but strong (white arrows) in cells expressing nsp11-K3779A (Fig 5D, middle and bottom panels). It is noteworthy that nsp11 in gene-transfected cells was distributed throughout the cell (Fig 5D, more fields can be observed in S6 Fig), while the distribution pattern was different in virus-infected cells (Fig 3E). This is because in virus-infected cells, nsp11 interacts with other viral proteins to form a replication complex whereas in gene-transfected cells, nsp11 exists independently without association with other viral proteins. Regardless of its cellular distribution, the mutational studies indicate that the reduction of MAVS by nsp11 was dependent on the functional EndoU activity.

### EndoU-mediated MAVS reduction exhibited impaired IFN signaling

**Fig 6:**
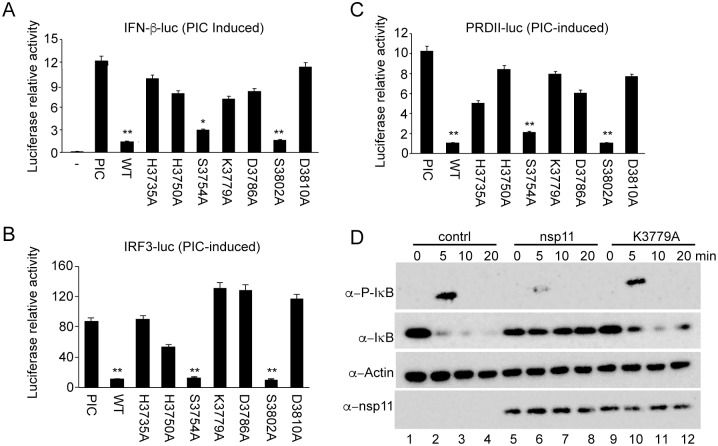
EndoU-mediated MAVS reduction exhibited impaired IFN signaling. (A-C) MARC-145 cells were co-transfected with pIFN-β-luc (0.5 μg) (A), or p4xIRF3-luc (0.5 μg) (B), or p(PRDII)_2_-luc (0.5 μg) (C), pRL-TK (0.05ug), and wild-type nsp11 (0.5 μg) or individual nsp11 EndoU mutant plasmids (0.5 μg/plasmid). At 24 h post transfection, cells were treated with poly(I:C) (0.5 μg/ml) for 16 h. Cells were lysed and examined for luciferase activity. The experiments were conducted in duplicate and repeated three times. Each sample expressing nsp11 was compared with poly(I:C) stimulated samples (*, P<0.05; **, P<0.01 by one-way ANOVA and bonferroni multiple comparison). (D) HeLa cells were co-transfected with empty vector control, or nsp11, or K3779A, and at 24 h, treated with 20 ng/ml of TNF-α for indicated time, followed by Western blot.

To examine whether the EndoU-dependent MAVS reduction led to the suppression of IFN-β production, nsp11 mutants were individually expressed in cells, and their ability to suppress IFN-β, IRF3, and NF-κB was investigated using pIFN-β-luc, p4xIRF3-luc, and p(PRDII)_2_-luc, respectively. While nsp11-WT, nsp11-S3754A, and nsp11-S3802A exhibited stronger suppression, nsp11-H3735A and nsp11-H3750A showed a relatively minor suppression (Fig 6A, 6B and 6C). The EndoU mutants whose endoribonuclease activity was lost also showed the loss of IFN suppressive activity. IFN bioassays were conducted for individual mutants to confirm the results of the reporter assays, and consistent results with reporter assays were obtained (S5 Fig). We further examined the activation of IRF3 and NF-κB using nsp11-K3779A as a representative mutant from the group of mutants that lost the EndoU activity in Fig 5. In nsp11-WT expressing cells, phosphorylated IRF3 was not seen as anticipated but was normally detected in nsp11-K3779A expressing cells (Fig 2B, top panel, lanes 4 and 6), indicating that the mutant nsp11-K3779A did not inhibit the IRF3 phosphorylation. For IκB phosphorylation, IκB MAb detected both phosphorylated (Fig 6D, top panel, upper bands) and unphosphorylated (lower bands) forms of IκB. When stimulated with TNF-α, IκB was rapidly degraded (Fig 6D, lane 2, lower band), and it was likely due to its phosphorylation and ubiquitination. In nsp11-WT expressing cells however, a lesser amount of phosphorylated IκB was identified (Fig 6D, lane 6 compared to lane 2, upper band), whereas the total amount of IκB was not decreased as fast as control (Fig 6D, lane 7 compared to lane 3, lower bands). This finding was consistent with the data obtained using phospho-IκB PAb (Fig 2C). In nsp11-K3779A expressing cells however, IκB was normally phosphorylated as anticipated after TNF-α stimulation, and a lesser amount of IκB was identified by 10 min post-stimulation (Fig 6D, lane 10 and 11), indicating that the nsp11-K3779A mutant did not block the IκB phosphorylation. Taken together, these results further support our findings that nsp11 antagonizes IFN-β expression via the EndoU-mediated reduction of MAVS expression level. Note that these conclusions were made heavily based on the data from the overexpression system.

### Essential requirements of EndoU activity for viral replication

**Fig 7:**
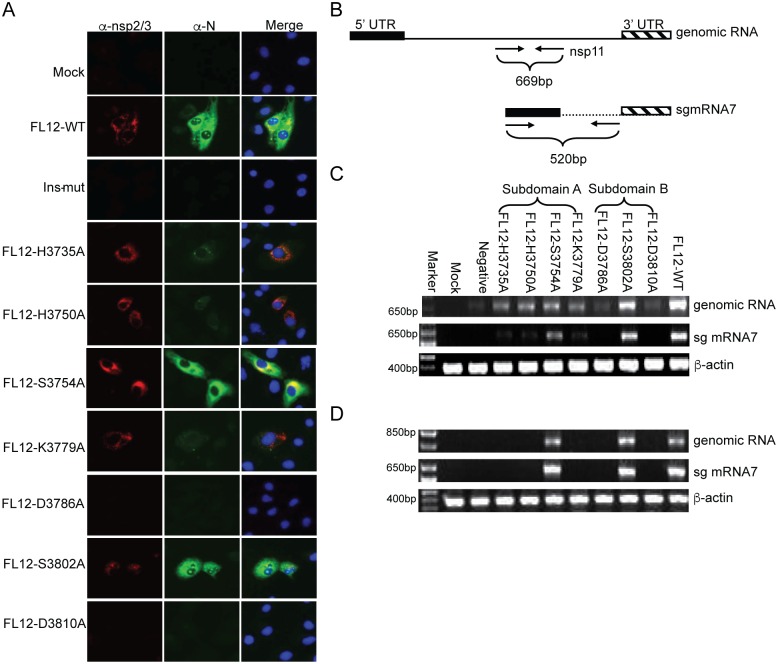
EndoU activity is essential for PRRSV replication. (A) MARC-145 cells were transfected with individual full-length viral genomic RNAs in vitro-synthesized from corresponding infectious cDNA clones using T7 RNA polymerase. At 16 h post-transfection, cells were co-stained with anti-nsp2/3 PAb and anti-N MAb (SDOW17) to examine the genome replication and sub-genomic (sg) mRNA7 transcription, respectively. All images were recorded under the identical exposure time and contrast/brightness settings. (B) Schematic representation of the primer positions and RT-PCR analysis that were used to detect the genomic RNA replication and sg mRNA7 transcription. (C) Detection of the ‘passage 1’ (P1) viral RNA in cells transfected with individual mutant genomic RNAs. Total cellular RNA was extracted at 16 h post-transfection and RT-PCR was conducted to amplify the region as illustrated in (B). β-actin was amplified as an internal control. (D) Detection of ‘passage 2’ (P2) viral RNA in cells inoculated with the P1 supernatant. Total cellular RNA was extracted at 6 days post-infection and subjected to RT-PCR.

In addition to the IFN antagonism of EndoU, we further examined whether the nsp11 EndoU activity would be essential for virus replication. A previous study suggested that EndoU-deficieny was not lethal for EAV but EndoU-deficient viruses were replication-compromised [35]. We constructed a series of EndoU mutant PRRSV genomic clones as indicated in Fig 4 using FL12 infectious cDNA clones. A stop-codon insertion mutant (Ins-mut) was additionally constructed to destroy the nsp11 gene and was used as a negative control. Cells were transfected with the full-length mutant genomic RNAs transcribed off their respective genomic clones, and PRRSV nsp2/3 and N proteins were examined by IFA at indicated times post-transfection (Fig 7A). To our surprise, two mutant genomes FL12-nsp11-D3786A and FL12-nsp11-D3810A did not express the nsp2/3 and N proteins in transfected-cells even after 72 h post-transfection (p.t.). Three mutant genomes FL12-nsp11-H3735A, FL12-nsp11-H3750A, and FL12-nsp11-K3779A showed a strong signal for nsp2/3 expression but a reduced signal for N protein compared to those of wild-type PRRSV at 16 h p.t. (Fig 7A). The numbers of cells positive for nsp2/3 expression for FL12-nsp11-H3735A, FL12-nsp11-H3750A, and FL12-nsp11-K3779A were increased by approximately 10% at 24 h p.t. compared to those at 16 h p.t. As anticipated, FL12-nsp11-S3754A and FL12-nsp11-S3802A expressed the nsp2/3 and N proteins at comparable levels to those of wild-type PRRSV.

To confirm the IFA results for nsp2/3 and N protein expressions, viral RNAs were examined in transfected cells at 16 h p.t. The viral genomic RNA replication was determined using nsp11-specific primers for a 669 bp fragment, and the sub-genomic (sg) mRNA7 transcription was determined using a forward primer within the 5’-UTR of the genomic RNA and a reverse primer for ORF7, which would produce a 520 bp fragment (Fig 7B). FL12-nsp11-S3802A was found to produce the genomic RNA and sg mRNA7 as with FL12-nsp11-WT (Fig 7C). However, FL12-nsp11-D3786A and FL12-nsp11-D3810A did not synthesize the genomic RNA and sg mRNA7. FL12-nsp11-H3735A, FL12-nsp11-H3750A, FL12-nsp11-S3754A, and FL12-nsp11-K3779A produced similar levels of PRRSV genomic RNA. However, three mutants FL12-nsp11-H3735A, FL12-nsp11-H3750A, and FL12-nsp11-H3779A produced much less amounts of sg mRNA7 compared to FL12-nsp11-S3754A (Fig 7C).

To determine whether progeny viruses were actually produced from the mutant genomic RNAs, the supernatants (passage 1) from genomic RNA-transfected cells were inoculated to fresh MARC-145 cells and their infectivity was monitored. Inoculation of FL12-nsp11-WT, FL12-nsp11-S3754A, and FL12-nsp11-S3802A developed extensive CPEs by 6 days post-infection, and the titers of these viruses were determined as 5.6x10^2^, 5.6x10, and 3.2x10^2^ TCID_50_/ml, respectively (Table 1). For FL12-nsp11-D3786A, FL12-nsp11-D3810A, FL12-nsp11-H3735A, FL12-nsp11-H3750A, and FL12-nsp11-K3779A, no CPE was developed even at 6 days post-infection. N-protein staining of cells inoculated with ‘passage 1’ virus of FL12-nsp11-WT, FL12-nsp11-S3754A, and FL12-nsp11-S3802A showed a specific signal at 6 days post-inoculation but no N-protein staining was detected for FL12-nsp11-H3735A, FL12-nsp11-H3750A, FL12-nsp11-K3779A, pFL12-nsp11-D3786A, and FL12-nsp11-D3810A. Production and release of infectious virus to the cell culture supernatants were also examined by RT-PCR using the viral RNA extracted from culture supernatants of cells inoculated with ‘passage 1’ virus, and the results confirmed the absence of the genomic RNA and sg mRNA7 syntheses by nsp11 mutants containing impaired EndoU activity: FL12-nsp11-H3735A, FL12-nsp11-H3750A, FL12-nsp11-K3779A, FL12-nsp11-D3786A, and FL12-nsp11-D3810A. Only two mutants FL12-nsp11-S3754A and FL12-nsp11-S3802A, and the wild-type clone FL12-nsp11-WT, were RT-PCR positive for both genomic RNA and sg mRNA7 (Fig 7D). These results demonstrate that the nsp11 EndoU activity was essential for virus infectivity and no progeny virus was produced when it was destroyed.

**Table 1:** Overview of genotype and viability of PRRSV EndoU mutant viruses.

Mutant virus	nsp11 mutation (wt → mutated sequence)	Passage 1 (P1)^a^				Passage 2 (P2)^a^		Viability^e^
		nsp2/3 IFA^b^	N IFA^b^	Log titre^c^ (16hr)	Log titre^c^ (24hr)	N IFA^d^	RT-PCR^d^	
FL-12 WT	none	+++	+++	2	2.75	+++	+	wild type
Defective	One nt insertion (CAT)→(CCAT)	-	-	-	-	-	-	nonviable
**Subdomain A**								
H3735A	His3735 (CAT)→Ala (GCT)	++	+	<10	<10	-	-	Low titer
H3750A	His3750 (CAC)→Ala (GCC)	++	+	<10	<10	-	-	Low titer
S3754A	Ser3754 (TCC)→Ala (GCC)	+++	+++	1	1.75	+++	+	viable
K3779A	Lys3779 (AAA) →Ala (GCA)	++	+	<10	<10	-	-	Low titer
**Subdomain B**								
D3786A	Asp3786 (GAT)→Ala (GCT)	-	-	-	-	-	-	nonviable
S3802A	Ser3802 (TCC)→Ala (GCC)	+++	+++	1.75	2.5	+++	+	viable
D3810A	Asp3810 (GAC)→Ala (GCC)	-	-	-	-	-	-	nonviable

^a^MARC-145 cells were transfected with genomic RNA transcripts. Cell culture supernatants and intracellular RNA were collected at indicated time points, and the progeny virus in the supernatants was designated as ‘passage 1’ (P1). Fresh MARC-145 cells were inoculated with P1 and the culture supernatant was designated as ‘passage 2’ (P2).

^b^Immunofluorescence assay (IFA) was conducted at 16 h post-transfection for P1. Percentage of IFA positive cells was indicated as +/-. ++++, greater than 12%; +++, 10%; ++, approximately 5%; + weakly positive in less than 2%.

^c^Virus titers were determined by end-point dilution on MARC-145 cells at 6 days post-infection of P1 virus and expressed as log 50% tissue culture infective dose per ml (TCID_50_ /ml).

^d^Immunofluorescence assay and RT-PCR for P2 were performed at 6 days post-infection.

^e^Nonviable indicates no detectable signal in P2 cells by IFA and RT-PCR.

## Discussion

Viruses have developed various mechanisms during evolution to modulate the IFN response to evade the innate immune defense of hosts. For PRRSV, both North American and European genotypes have been shown to produce unusually low levels of type I IFNs during infection in cells and pigs [42], and different isolates suppress varying degrees of IFNs [43]. VR-2332 and NVSL 97–7895 (FL12) strains in particular have been shown to possess a potent ability of IFN suppression. Viruses may carry more than one protein to modulate IFN response [44–46], and for PRRSV, nsp1α, nsp1β, nsp2, nsp4, and N proteins have been demonstrated to antagonize the NF-κB or IFN pathways [14–19]. In the present study, we have further investigated nsp11-regulated IFN suppression and identified a mechanism interfering with the RLR mediated signaling by nsp11. PRRSV nsp11 inhibited the phosphorylation of both IRF3 and IκB, and thus blocked their activation and nuclear translocation. It is intriguing that the mRNA of both RIG-I and MAVS, which is a critical adaptor within the RIG-I/MDA-5 signaling, was decreased by nsp11, and as a result, MAVS protein was significantly reduced. Furthermore, this reduction was EndoU-dependent as shown by mutational studies. H3735A, H3750A, and K3779A mutants, whose RNase catalytic sites were mutated, showed the loss of IFN suppression and normal level expression of MAVS. In the case of EAV nsp11, a double mutation of H3735A and H3750A caused the loss of RNase activity and it is postulated that the single mutation at one of the two residues may result in a partial RNase activity [41]. This may explain why H3735A and H3750A showed a partial suppression of IFN-β activity. The aspartic acid at D3786 and D3810 may play a role in stabilizing the secondary structure of nsp11 as seen for nsp15 of SARS-CoV [30], which is the PRRSV nsp11 homolog in coronaviruses. Thus, D3786A and D3810A mutant genes that lost their suppressive function may be due to an incorrect folding/assembly of the nsp11 complex. S3754A and S3802A retained the suppressive function for IFN-β production and reduced MAVS mRNA as anticipated, as with wild-type nsp11. Taken together, our data, although majorly carried out in the context of transfection, showed that mRNAs of both RIG-I and MAVS were reduced by the nsp11 RNase activity, and therefore the RLR signaling failed to activating IRF3 and NF-κB, leading to the suppression of type I IFN.

In fact, nsp11 is not the only viral protein in PRRSV targeting MAVS and RIG-I. A recent study reports that nsp4, a 3C-like viral proteinase, cleaves MAVS protein into small products and thus leads to the abolishment of IFN production [19]. A similar strategy is also utilized by hepatitis C virus [47], and so it seems clear that many viruses often target not only proteins but also RNAs to counteract antiviral responses of hosts. The SARS-CoV nsp1 protein binds to the 40S ribosome to inactivate translation and also induces a template-dependent cleavage of mRNA [48]. The herpes simplex virus VHS protein degrades mRNA by its endoribonuclease activity and also affects translation by interacting with eIF4F [49, 50]. The influenza virus NS1 protein targets intracellular viral RNA and prevents binding to RIG-I and PKR, resulting in the inhibition of IRF3 activity [51–54]. The E^rns^ protein of bovine viral diarrhea virus is an exoribonuclease targeting extracellular RNAs, and the cleavage of RNA by E^rns^ prevents the activation of TLR3 and blocks its downstream signaling [39]. Since PRRSV nsp11 is an endoribonuclease, the reduction of MAVS mRNA by nsp11 is postulated to be due to the mRNA degradation by endoribonuclease activity. In our study, nsp11 did not reduce the luciferase activity of the control plasmid (S3 Fig), and furthermore, the total amounts of IRF3 and p65 were not decreased in nsp11-expressing cells, whereas the both phosphorylated IRF3 and IκB were reduced, ruling out the possibility of non-specific degradation of RNA. Indeed, both RIG-I mRNA and MAVS mRNA were reduced by nsp11, whereas IRF3 mRNA and p65 mRNA were not affected (Fig 3F), suggesting that the mRNA reduction caused by nsp11 was rather selective. So far, from this study, we have identified two targets of nsp11, RIG-I and MAVS. We cannot rule out the existence of other possible substrates of nsp11.

It is noted that the cellular distribution of nsp11 in PRRSV-infected cells was restricted to the perinuclear region, whereas in nsp11-transfected cells, nsp11 was widely expressed throughout the cytoplasm. The restricted distribution of nsp11 in infected cells may be due to its participation in viral replication and may also minimize the nsp11 toxicity by preventing massive degradation of viral and cellular RNAs. Regardless of the cellular distribution, nsp11 retained its ability to decrease the MAVS gene expression, implying the specific reduction of MAVS mRNA by nsp11 in PRRSV-infected cells.

In our study, the nsp11 mutant genomic clones were constructed so that the type I IFN suppression by nsp11 could be studied using infectious viruses. FL12-nsp11-H3735A, FL12-nsp11-H3750A, and FL12-nsp11-K3779A were EndoU-knock-out mutants, and they appeared to be non-viable. Although these mutant genomic clones were positive for N-protein staining in transfected cells, no CPE was observed when the culture supernatants were passaged to fresh MARC-145 cells, indicating the absence of infectivity. Two Asp mutant clones FL12-nsp11-D3786A and FL12-nsp11-D3810A were totally negative for the N and nsp2/3 proteins in genomic RNA-transfected cells as well as culture supernatant-inoculated cells, indicating that these mutations were also lethal for infectivity and non-viable. The data from the mutant genomic clones were partially consistent with EAV nsp11 mutant viruses and SARS-CoV nsp15 mutant viruses, with an exception for mutant viruses of three catalytic-site mutations, FL12-nsp11-H33735A, FL12-nsp11-H3750A, and FL12-nsp11-K3779A. [26, 35, 55]. Nevertheless, our data indicate that the EndoU domain of PRRSV nsp11 plays a critical role for virus replication and is involved in the processing of viral RNA synthesis. Our findings contribute to our understanding on the molecular mechanisms of innate immune evasion utilized by PRRSV.

## Materials and Methods

### Cells and viruses

MARC-145 (a subline of African green monkey kidney MA-104 cells; [56]) and HeLa (NIH AIDS Research Reference Reagent Program, Germantown, MD) cells were maintained in Dulbecco’s modified Eagle’s medium (DMEM; Mediatech Inc., Manassas, VA) containing 10% heat-inactivated fetal bovine serum (FBS; Hyclone, Logan, UT) in a humidified incubator with 5% CO_2_ at 37°C. Baby hamster kidney (BHK-21) cells were maintained in modified Eagle’s medium (MEM; Mediatech Inc.) supplemented with 10% heat-inactive FBS. PRRSV strain NVSL 97–7895 (FL12) was generated from its infectious clone pFL12 [57] which was propagated in MARC-145 cells. Recombinant vesicular stomatitis virus expressing green fluorescent protein (VSV-GFP) was kindly provided by Adolph Garcia-Sastre (Mt. Sinai School of Medicine, New York, NY). VSV-GFP was propagated in BHK-21 cells. And it was titrated in MARC-145 cells using a standard plaque assay.

### DNA cloning

The PRRSV nsp11 coding sequence was amplified by PCR using the FL12 infectious clone and specific primers listed in Table 1. The PCR fragment was cloned into pCMV-Tag1 (Strategene, La Jolla, CA) using the Bam HI and Xho I. The nsp11 gene which fused with the N-terminal Flag tag was digested with Bam HI and Xho I.The gene was ligated in the mammalian expression vector pXJ41 [58], which was then named pXJ41-nsp11 in this study. The reporter plasmids p4xIRF3-luc and pIFN-β-luc were provided by Stephen Ludwig (Institute of Molecular Medicine, Heinrich-Heine-Universitat, Germany). The p4xIRF3-luc plasmid contains the luciferase gene in front of four copies of positive regulatory domain I/III (PRD I/III) of the IFN-β promoter region. The pIFN-β-luc plasmid contains the luciferase reporter gene which was placed under the IFN-β promoter. The plasmid pluc-(PRDII)2 contains two copies of the NF-κB binding region PRDII of the IFN-β promoter in front of the luciferase gene which was provided by Stanley Perlman (University of Iowa, Iowa city, IA) [59]. The pTATA-luc construct was placed under the control of TATA-box promoter as a negative control. pRL-TK (Promega, Madison, Wisconsin) for renilla-luciferase expression under the control of the herpes simplex virus thymidine kinase promoter was used to normalize transfection efficiencies. The pcDNA3-RIG-I and pcDNA3-MAVS plasmids were obtained from Joanna Shisler (University of Illinois at Urbana-Champaign, Urbana, IL). pcDNA3-RIG-I contains the functionally activated caspase activation and recruit domain (CARD) region whichis constitutively active.

### Antibodies

Polyinosinic:polycytidylic acids (poly [I:C]), anti-Flag MAb M2, and anti-MAVS polyclonal Ab (PAb) were purchased from Sigma (St. Louis, MO). Anti-β-actin MAb, anti-IRF3 PAb, anti-human heat shock protein 90 (HSP90) MAb, and anti-human poly[ADP-ribose] polymerase protein (PARP) PAb were purchase from Santa Cruz Biotechnologies Inc. (Santa Cruz, CA). PAbs for phosphor-IRF3 (Ser396) and TNFα were purchased from Cell Signaling (Danvers, MA). The peroxidase-conjugated goat anti-mouse IgG and fluorescein (FITC)-conjugated goat anti-mouse IgG were purchased from Jackson ImmunoResearch (West Grove, PA). The goat anti-rabbit antibody conjugated with Texas red and goat anti-mouse antibody conjugated with Alexa green were purchased from Invitrogen (Carlsbad, CA). The nsp11-specific rabbit antibody was generated as follow. Since wild-type PRRSV nsp11 was toxic to E. coli [41], one of the EndoU mutants K3779A was subcloned into the pET-28a(+) expression vector containing a His tag at the C-terminus. This plasmid was transformed into E. coli BL21. PRRSV nsp11 protein expressed in E. coli was purified using the HisTrap column to 1 mg/ml according to the manufacturer’s instruction (GE Healthcare life science, Piscataway, NJ). A total of 2 mg of proteins was immunized to a rabbit.Rabbit anti-PRRSV-nsp11 polyclonal antibody was generated at the Immunological Research Center (University of Illinois, Urbana, IL). Serum was collected periodically which was examined for specific antibody using immunoblot and immunofluorescence assays.

### Dual luciferase assay

Both HeLa and MARC-145 cells were used to examine luciferase reporter activities. IFN stimulation was conducted using poly(I:C). Cells were seeded in 12-well plates.In each well, 0.05 μg of pRL-TK, 0.5 μg of pIFN-β-luc, p4xIRF3-luc, or pTATA-luc, and 0.5 μg of pXJ41-nsp11 were co-transfected using Lipofectamine 2000 according to the manufacturer’s instruction (Invitrogen). Twenty-four hours after DNA transfection, 0.5 μg of poly(I:C) was transfected into cells grown in 6-well plate for stimulation for 16 h. Cells were lysed using Passive lysis buffer (Promega).Supernatants were measured for luciferase activity using the Dual luciferase reporter assay system (Promega) in a luminometer (Wallac 1420 VICTOR multi-label counter, Perkin Elmer, Waltham, MA). For stimulation by RIG-I, MAVS, or IRF3-GFP, 0.5 μg of the constitutively active form of pcDNA3-RIG-I, pcDNA-MAVS, or pGFP-N1-IRF3 was co-transfected with a reporter plasmid into cells. Forty hours post-transfection, cells lysates weremeasured for luciferase activity which was then normalized using renilla luciferase activities according to the manufacture’s instruction (Promega).

### VSV-GFP bioassay

Culture supernatants collected from cells prepared for luciferase assays were diluted to make two-fold serial dilutions. Fresh MARC-145 cells were prepared in 96-well plates and incubating with each dilution of supernatants for 24 h. Then followed by infection with VSV-GFP at an MOI of 0.1. GFP expression was then visualized by fluorescent microscope at 16 h post-infection. Amounts of IFN in the supernatants were presented as the inhibitory ability for VSV-GFP replication in MARC-145 cells.

### Immunofluorescence assay (IFA)

HeLa cells were grown on coverslips for 16 h. Cells were transfected with 2 μg of pXJ41-nsp11 or its derivatives for 24 h. For IRF3 nuclear translocation, cells were transfected with 1 μg of poly(I:C) for 6 h. For p65 nuclear translocation, cells were stimulated by incubation with 20 ng/ml of TNF-α for 1 h at 24 h post-transfection. Cells were fixed with 4% paraformaldehyde in phosphate buffered saline (PBS) at room temperature (R/T) for 15 min, followed by permeabilization for 10 min using 0.1% Triton X-100 in PBS at RT. After washing 3 times with PBS, cells were incubated with anti-Flag MAb (1:600), together with anti-IRF3 PAb (1:200) or anti-p65 PAb (1:100) for 1 h at R/T. Following 3 washes with PBS, cells were incubated with goat anti-rabbit antibody conjugated with Texas red (1:500) or goat anti-mouse antibody conjugated with Alexa green (1:800) for 1 h. Nuclei were stained with 4, 6-diamidino-2-phenylindole (DAPI, Molecular Probes Inc., Eugene, OR). For co-staining of nsp2/3 and N proteins in PRRSV-infected MARC-145 cells, cells were stained with rabbit nsp2/3 specific PAb (1:2000; obtained from E. J. Snijder, Leiden University Medical Center, Leiden, Netherlands) and SDOW17 MAb (1:200). After staining, coverslips were mounted on microscope slides in the mounting buffer (60% glycerol and 0.1% sodium azide in PBS).And the slides were visualized using a fluorescence microscope (Laborlux 12, Leitz).

### Immunoblotting

Cells were lysed in the RIPA buffer (50 mM Tris/HCl [pH 8], 150 mM NaCl, 1% NP-40, 1% SDS, 0.5% sodium deoxycholate) containing 1 mM PMSF (phenylmethanesulphonyl fluoride). Insoluble materials were removed by centrifugation at 4°C for 10 min at 12,000 rpm in a microcentrifuge.And cell lysates were resolved by 10% SDS-PAGE followed by transferred to Immobilon-P membrane (Millipore). After blocking with 5% skim milk powder dissolved in TBS-T (10 mM Tris-HCl [pH 8.0], 150 mM NaCl, 1% Tween 20) for 1 h at R/T, membranes were incubated with the primary antibody in TBS-T containing 5% skim milk or 5% BSA at 4°C overnight. After 5 washes with TBS-T, the horseradish peroxidase-conjugated secondary antibody was incubated with the membrane for 1 h at R/T. Membranes were washed 5 times again. And proteins were visualized using the ECL detection system (Thermo, Minneapolis, MN)

### Cell fractionation

HeLa cells were grown in 6-well plates to 80% confluency and were transfected with 2 μg of pXJ41-nsp11 each well for 24 h. Cells were stimulated by transfection with 1 μg of poly(I:C) per well for 6 h and fractionated using the nuclear/cytosol fractionation kit according to the manufacturer’s instructions with minor modifications (BioVision Technologies, Exton, PA). Briefly, cells were collected in 800 μl of PBS per well. And the cells were then centrifuged at 4°C for 5 min at 600 x g in a microcentrifuge. Cell pellets were resuspended in CEB (cytosolic extraction buffer)-A, and incubating for 10 min on ice prior to addition of CEB-B. The lysates were centrifuged at 4°C for 5 min at 12,000 rpm in a microcentrifuge. And the supernatants were kept as the cytoplasmic fraction. The nuclear pellet was resuspended in NEB (nuclear extraction buffer). And vortexed the cells for 30 s. This step was repeated 5 times 10 min each. The nuclear pellet was centrifuged at 4°C for 10 min at 12,000 rpm in a microcentifuge. And the supernatants were kept as the nuclear fraction. The cytoplasmic and nuclear fractions were resolved by SDS-PAGE followed by immunoblotting.

### Quantitative PCR

Real-time PCR was performed using ABI Sequence Detector System (ABI Prism 7000 Sequence Detection System and software; Applied Biosystems) in a final volume of 25 μl containing 2.5 μl of cDNA from the reversed transcribed reaction, primer mix (2.5 pM each of sense and antisense primers), 12.5 μl of SYBR Green Master Mix (Applied Biosystems) and 5 μl of distilled water. The oligonucleotide primers were designed using Vector NTI software (Invitrogen) or obtained from literatures (S1 Table). The amplification parameters were 40 cycles of two steps each cycle comprised of heating to 95°C and 60°C. The final mRNA levels of the genes were normalized using GAPDH.

### Aqua Live/Dead staining and flow cytometry

MARC-145 cells grown in 6-well-plate were transfected with 2 microgram pXJ41 and nsp11 WT or nsp11 mutants using Lipofectamine 2000. At 24 hours post transfection, cells were trypsinized and suspending in 1ml PBS with 1% BSA (PBSA). Cells were then washed once with PBSA. Then resuspened the cells in 500 ml PBSA. 2ul of Aqua reagent was added to each sample with incubating in ice for 10 min. Then wash the cells with PBSA two times. Cells were then suspension in 1ml PBSA before applying to BD RSLII flow cytometry.

### Site-directed mutagenesis of nsp11 and construction of mutant PRRSV

Mutations in the nsp11 EndoU domain were made using primers (Table 1). And the mutations were introduced into the PRRSV infectious clone pFL12 by PCR. PCR was performed using 5 μl of 10x buffer (500 mM KCl, 100 mM Tris-HCl PH 9.0, 1% Triton-X, 2 mM Mg^2+^, and H_2_O), 200 ng of the FL12 infectious clone, 125 ng each of forward and reverse primers, 1 unit of Pfu, and 1 μl of dNTP (25 mM each NTP) in a 50 μl reaction. The amplification was carried out under the following conditions: 95°C for 2 min, followed by 18 cycles of 95°C for 1 min, 58°C for 50 sec and 68°C for 22 min ending with 68°C for 7 min. PCR products were digested with 1 unit Dpn I for 1 h. And the products were transformed into XL Golden E. coli cells (QuikChange II XL Site-Directed Mutagenesis kit; Stratagene, La Jolla, CA). Desired nucleotide changes and respective primers were listed in S1 Table. An out-of-frame nsp11 defective mutant (Ins-mut) was made by inserting an additional ‘C’ immediately upstream of the first nucleotide of the H3735 codon.

### Transfection of in vitro-synthesized genomic RNA and generation of PRRSV mutants

The PRRSV full-length infectious clone pFL12 and its mutant derivatives were linearized with Acl I as a template for *in vitro* transcription of capped RNA using the mMESSAGE mMACHINE Ultra T7 kit according to the manufacture’s instruction (Invitrogen). RNAs were precipitated with LiCl. And pellets were resuspended in 20 μl RNase-free water. Transfection was performed in MARC-145 cells using the Nucleofector device (Amaxa; Lonza Walkersville Inc., Walkersville, MD). Approximately, 2×10^7^ cells were trypsinized, with PBS washing, and resuspending in the Nucleofector solution T. Approximately 2×10^6^ cells in 0.1 ml of cell suspension was used for one transfection. For transfection, 7 μg of RNA transcript was added to the cell suspension, which was then electroporated using the Amaxa program K-29. After electroporation, cells were diluted in 10 ml of DMEM. And cells were seeded in 6-well plates. The supernatants were collected at 16, 24, 48, and 72 h post-transfection. And progeny viruses were recovered and designated as ‘passage 1’ (P1). P1 virus was transferred to fresh MARC-145 cells. And the virus was incubated for 6 days, followed by collection of supernatants (designed as ‘passage 2’ (P2)). Cytopathic effect (CPE) was monitored daily. IFA and RT-PCR were performed at 16 h post-transfection and 6 day post-infection. P1 and P2 supernatants were titrated by an endpoint dilution assay. And progeny virus titers were expressed as tissue culture infective dose 50 (TCID_50_).

### Detection of intracellular viral RNA

Intracellular viral RNA was extracted from cells using Trizol according to the manufacture’s instruction (Invitrogen). The reverse primer nsp11-R (genomic nt positions 11593–11611; 5’-TTCAAGTTGAAAATAGGC-3’) or ORF7-R (genomic nt positions 15197–15219; 5’- TGATGCGTCGGCAAACTAAACTC-3’) was used for reverse transcription, followed by PCR amplification using the forward primer nsp11-F (genomic nt positions 10943–10962; 5’-GGGTCGAGCTCCCCGCTCCC-3’) or 5-UTR-F (genomic nt positions 1–26; 5’-ATGACGTATAGGTGTTGGCTCTATGC-3’). The primer pairs produce either a 669 bp fragment from the genomic RNA or a 520 bp fragment from subgenomic (sg) mRNA7. The β-actin gene was amplified using actin-F (5’- GCGCGGCTACAGCTTCACCAC-3’) and actin-R (5’- GGGCGCCAGGGCAGTAATCTC-3’) to generate a 382 bp fragment.

### Statistical analysis

Statistical analyses were performed using GraphPad Prism version 5.00 (San Diego California USA, (www.graphpad.com).

### Genes NCBI ID numbers and viral GenBank accession numbers

GAPDH, 2597; MAVS, 57506; p65, 5970; IRF3, 3661; EAV, NC_002532; LDV, NC_003092; SHFV, DQ489311; PRRSV isolates: FL12, NC_002534; VR2332, EF536003; 16244B, AF046869; CH-1a, AY032626; HB-1, AY150312; HB-2, AY262352; HN1, AY457635; HuN, EF517962; JX0612, EF488048; JXA1, EF112445; JXwn06, EF641008; LMY, DQ473474; MLV, EF484033; MN184, EF484031; MN184B, DQ176020; SD01, AY545985; NVSL-97-798, AY545985; P129, AF494042; PA8, AF176348; pL97-1, AY585241; PrimePac, DQ779791; SHH, EU106888; SY0608, EU144079; Wuh1, EU187484; Lelystad, M96262; 01CB1, DQ864705; EuroPRRSV, AY366525; HKEU16, EU076704.

## Supporting Information

S1 Fig Inhibition of IRF3 nuclear translocation. (TIF)

S2 Fig Inhibition of NF-κB nuclear translocation by nsp11. (TIF)

S3 Fig Absence of effects on TATA luciferase activity by PRRSV nsp11. (TIF)

S4 Fig RT-PCR for MAVS mRNA in nsp11-expressing and PRRSV-infected cells. (TIF)

S5 Fig VSV-GFP bioassay for nsp11 mutants. (TIF)

S6 Fig Reduction of MAVS expression in {RRSV-infected cells and nsp11 gene-transfected cells. (TIF)

S1 Table Oligonucleotides and their sequences. (DOCX)
