# Supplementary figures and images for: Nonstructural Protein 11 of Porcine Reproductive and Respiratory Syndrome Virus Suppresses Both MAVS and RIG-I Expression as One of the Mechanisms to Antagonize Type I Interferon Production
Source: PLoS One. 2016 Dec 20;11(12):e0168314. doi: 10.1371/journal.pone.0168314 (PMC5172586; doi:10.1371/journal.pone.0168314)

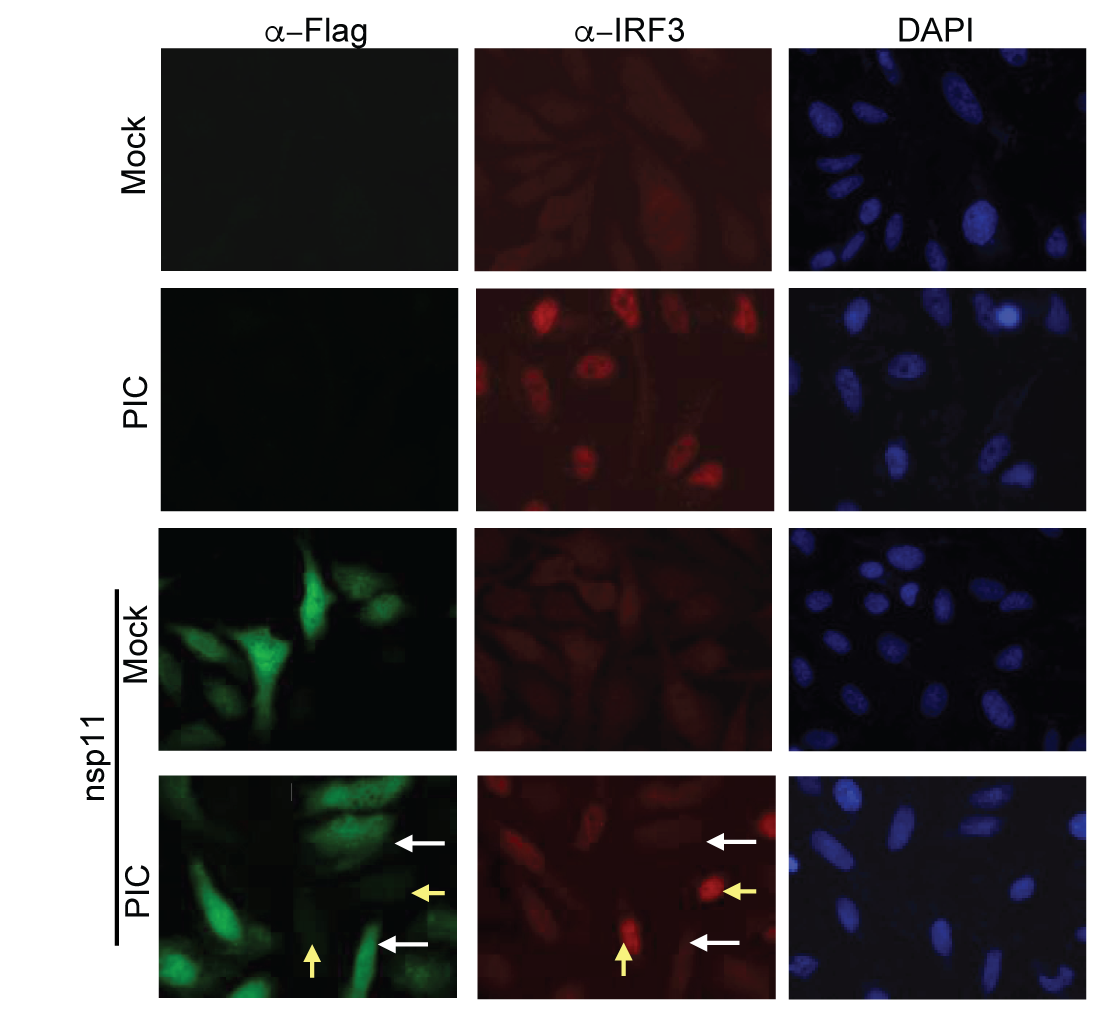

Supplement: S1 Fig — (TIF) [file pone.0168314.s001.tif]

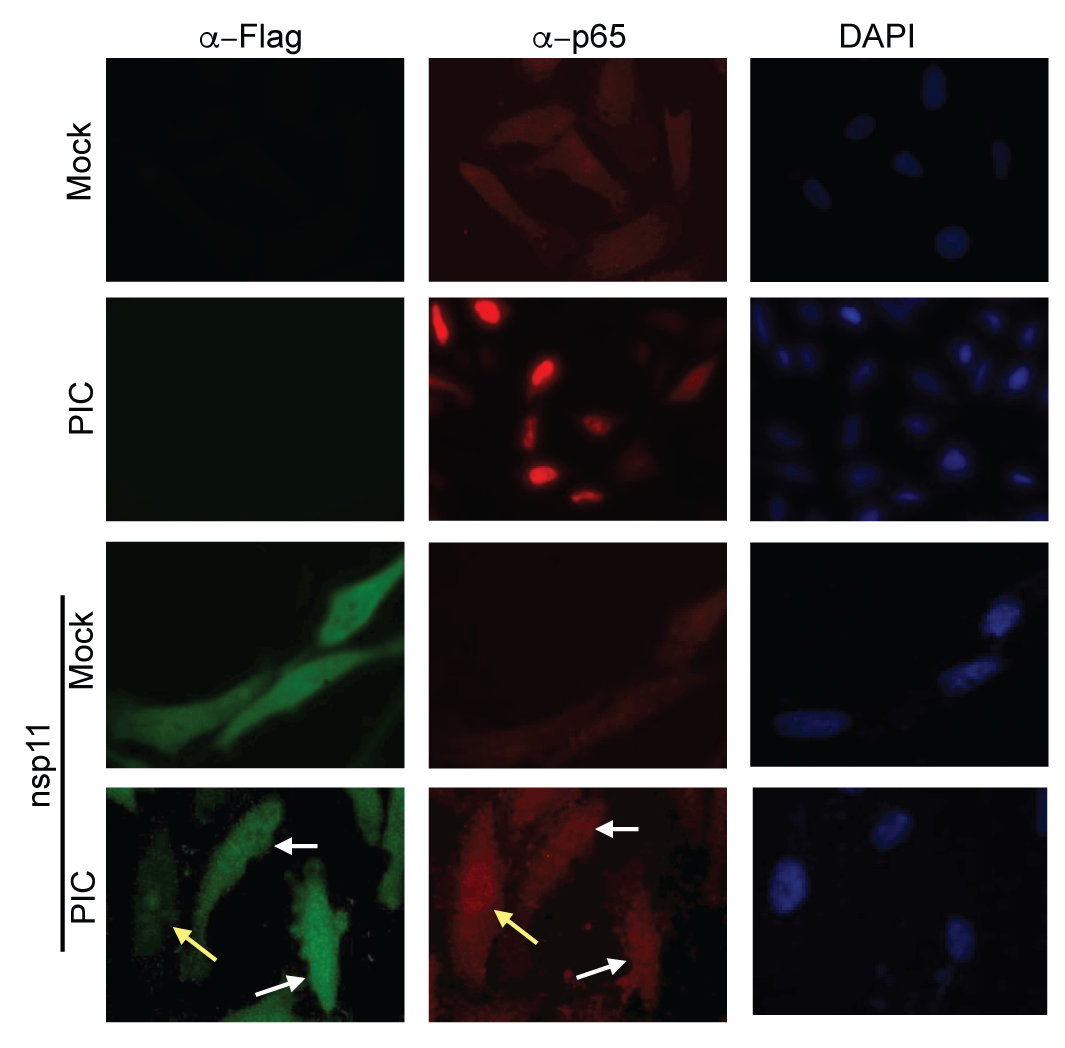

Supplement: S2 Fig — (TIF) [file pone.0168314.s002.tif]

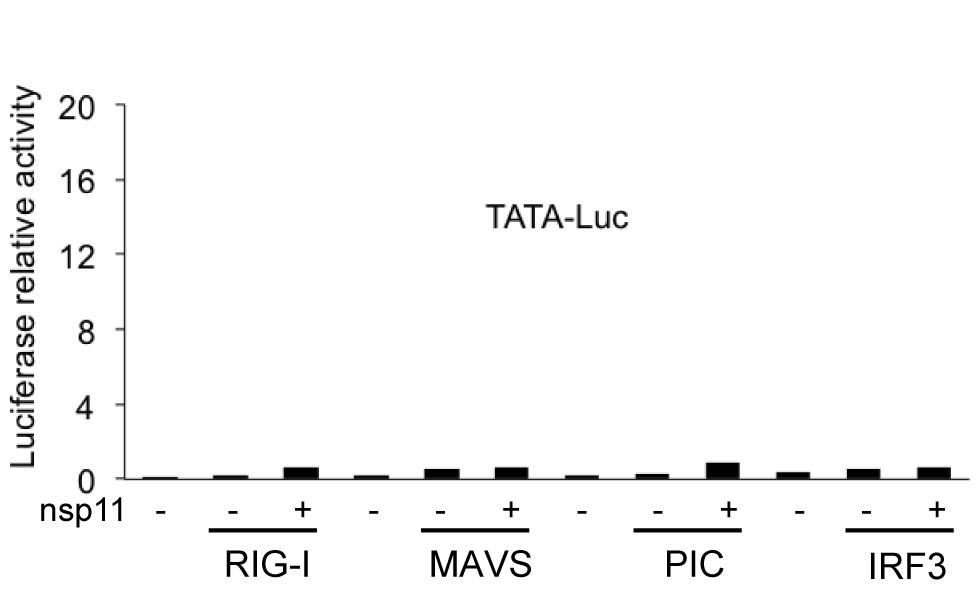

Supplement: S3 Fig — (TIF) [file pone.0168314.s003.tif]

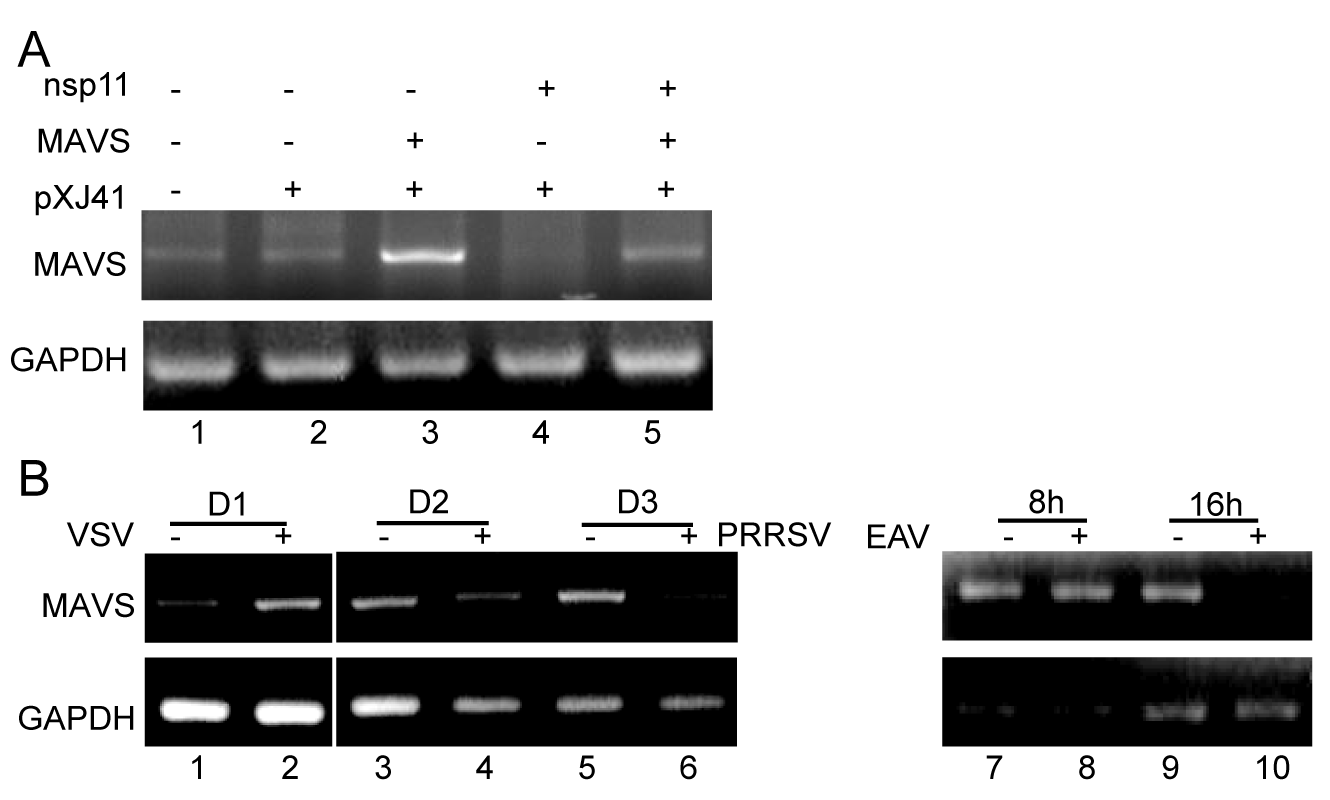

Supplement: S4 Fig — (TIF) [file pone.0168314.s004.tif]

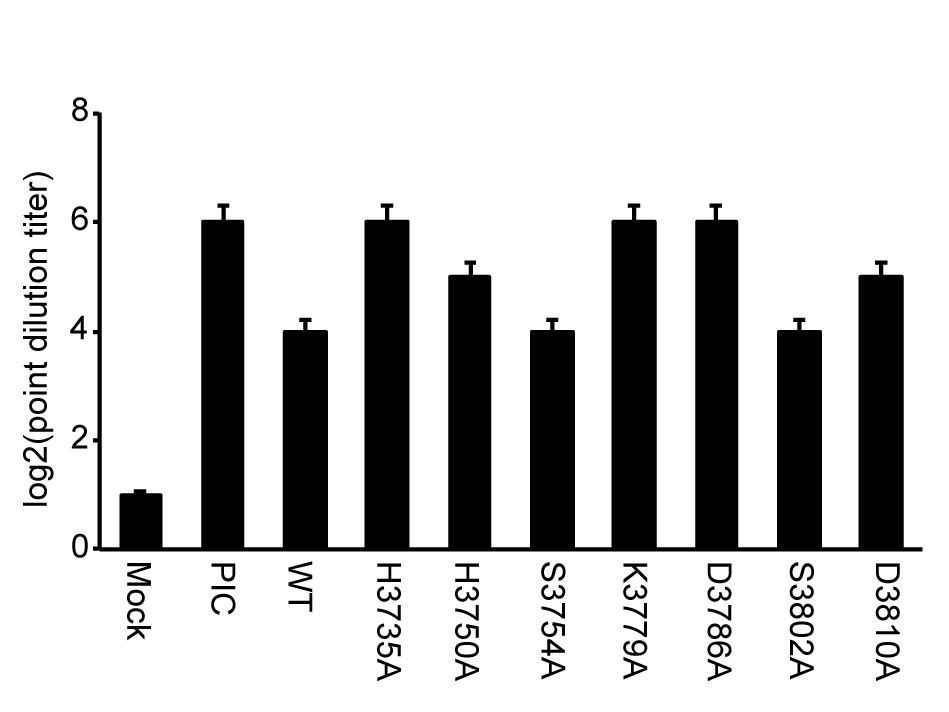

Supplement: S5 Fig — (TIF) [file pone.0168314.s005.tif]

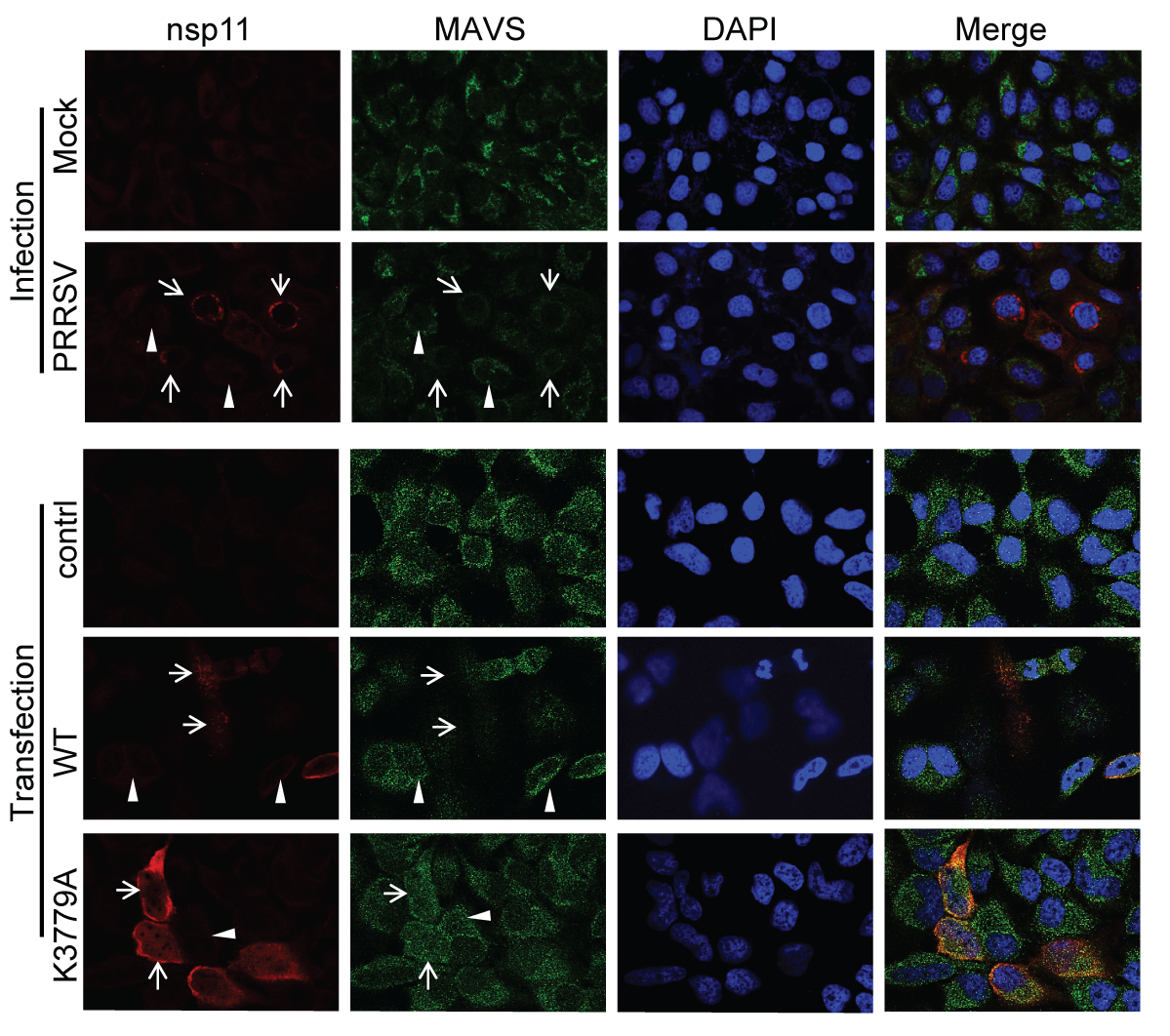

Supplement: S6 Fig — (TIF) [file pone.0168314.s006.tif]
