# Supplementary material for: Nonstructural Protein 11 of Porcine Reproductive and Respiratory Syndrome Virus Suppresses Both MAVS and RIG-I Expression as One of the Mechanisms to Antagonize Type I Interferon Production
Source: PLoS One. 2016 Dec 20;11(12):e0168314. doi: 10.1371/journal.pone.0168314 (PMC5172586; doi:10.1371/journal.pone.0168314)
Supplement: S1 Table — (DOCX) [file pone.0168314.s007.docx]

**SUPPLEMENTAL INFORMATION**

**S1 Table. Oligonucleotides and their sequences**

| **Oligonucleotides** | **Primer sequence** |
| --- | --- |
| nsp11-F | 5'-AAAGGATCCATATGGGGTCGAGCTCCCCGCTCCC-3' |
| nsp11-R | 5'-CCCCTCGAGTTCAAGTTGAAAATAGGC-3' |
| nsp11-H3735A-U | 5’-GAGTCCCTCCCCGCTGCTTTCATTGGC-3’ |
| nsp11-H3735A-L | 5’-GCCAATGAAAGCAGCGGGGAGGGACTC-3’ |
| nsp11-H3750A-U | 5’-CGTTGGAGGATGTGCCCATGTCACCTC-3’ |
| nsp11-H3750A-L | 5’-GAGGTGACATGGGCACATCCTCCAACG-3’ |
| nsp11-S3754A-U | 5’-CCATGTCACCGCCAAATACCTTCCG-3’ |
| nsp11-S3754A-L | 5’-CGGAAGGTATTTGGCGGTGACATGG-3’ |
| nsp11-K3779A-U | 5’-GGAAAGCCGCAGCAGCAGTTTGCAC-3’ |
| nsp11-K3779A-L | 5’-GTGCAAACTGCTGCTGCGGCTTTCC-3’ |
| nsp11-D3786A-U | 5’-GTTTGCACATTAACAGCTGTGTATCTCCCAG-3’ |
| nsp11-D3786A-L | 5’-CTGGGAGATACACAGCTGTTAATGTGCAAAC-3’ |
| nsp11-S3802A-U | 5’-CCAGAGACCCAGGCCAAGTGCTGG-3’ |
| nsp11-S3802A-L | 5’-CCAGCACTTGGCCTGGGTCTCTGG-3’ |
| nsp11-D3810A-U | 5’-GAAAATGATGTTGGCCTTCAAGGAAGTTCG-3’ |
| nsp11-D3810A-L | 5’-CGAACTTCCTTGAAGGCCAACATCATTTTC-3’ |
| nsp11-Sab-D-U | 5’-GGACTCAGCAATTTCTCGCTCCC-3’ |
| nsp11-Sab-D-L | 5’-GAAGTTCGACTGATGGTCTGGAAG-3’ |
| nsp11-Sa-D-L | 5’-CTAACAGATGTGTATCTCCCAGATCTCG-3’ |
| nsp11-Sb-D-U | 5’-GGTGCAAACTGCTTTTGCGG-3’ |
| hIPS-1-F | 5’-CGGGCATCAGGAGCAGGACA-3' |
| hIPS-1-R | 5'-TCACAGGCATCAAGGTGGTAGGC-3' |
| hRIG-I-F | 5'-AAACCAGAGGCAGAGAAGAGCAA-3' |
| hRIG-I-R | 5'-TCGTCCCATGTCTGAAGGCGTAAA-3' |
| hp65-F | 5'-CTGCAGTTTGATGATGAAGA-3' |
| hp65-R | 5'-TAGGCGAGTTATAGCCTCAG-3' |
| hIRF3-F | 5'-ACCAGCCGTGGACCAAGAG-3' |
| hIRF3-R | 5'-TACCAAGGCCCTGAGGCAC-3' |

Underlines indicate restriction enzyme recognition sequences used for cloning.
